# Graphene/Ag–Ag_2_S based hybrid nanostructure for methylene blue degradation

**DOI:** 10.3389/fchem.2025.1695385

**Published:** 2025-11-28

**Authors:** Talia Tene, Lala Gahramanli, Mustafa Muradov, MahammadBaghir Baghirov, Goncha Eyvazova, Stefano Bellucci, Jessica Alexandra Marcatoma Tixi, Cristian Vacacela Gomez, Rana Khankishiyeva, Lorenzo S. Caputi, Salvatore Straface

**Affiliations:** 1 Department of Chemistry, Universidad Técnica Particular de Loja, Loja, Ecuador; 2 Nano Research Laboratory, Center of Excellence, Baku State University, Baku, Azerbaijan; 3 Faculty of Physics, Chemical Physics of Nanomaterials, Baku State University, Baku, Azerbaijan; 4 National Institute of Materials Physics, Bucharest-Magurele, Romania; 5 Carrera de Estadística, Facultad de Ciencias, Escuela Superior Politecnica de Chimborazo (ESPOCH), Riobamba, Ecuador; 6 Department of Environmental Engineering (DIAm), University of Calabria, Rende, Italy; 7 Universidad ECOTEC, Samborondón, Ecuador; 8 Institute of Radiation Problems, Ministry of Science and Education of the Republic of Azerbaijan, Baku, Azerbaijan; 9 Azerbaijan University of Architecture and Construction, Baku, Azerbaijan; 10 UNICARIBE Research Center, University of Calabria, Rende, Italy; 11 Surface Nanoscience Group, Department of Physics, University of Calabria, Rende, Italy

**Keywords:** graphene, Ag–Ag2S NWs, MB dye, photocatalytic degradation, degradation efficiency

## Abstract

In this study, novel 2D/1D graphene/silver-silver sulfide (Ag–Ag_2_S) hybrid nanocomposites were successfully synthesized and characterized using X-ray Diffraction (XRD), Ultraviolet-Visible (UV-Vis) spectroscopy, Fourier-transform infrared spectroscopy (FTIR), and Scanning Electron Microscopy (SEM). The structural–optical properties and dye-photodegradation performance of Ag nanowires (NWs), Ag–Ag_2_S core–shell NWs, and a 2D/1D graphene/Ag–Ag_2_S hybrid nanocatalyst were examined. SEM confirms uniform, non-agglomerated Ag NWs and a layered graphene morphology; after sulfidation, Ag_2_S (and incidental Ag_2_O) forms on Ag NW surfaces, while Ag–Ag_2_S NWs are randomly distributed across graphene sheets. XRD results confirm the presence of crystalline phases corresponding to Ag, Ag_2_S, and silver oxide (Ag_2_O), indicating successful hybridization and partial oxidation during synthesis. UV–Vis spectra show the two Ag localized surface plasmon resonances (LSPR) (∼350/380 nm) collapsing into a broadened band upon Ag_2_S shelling, consistent with higher dielectric loss and interfacial damping; graphene/Ag–Ag_2_S is dominated by a π–π* transition near 200–250 nm. Tauc analysis yields, E.g., ≈ 2.9 eV (Ag NWs), and after hybridization, approximately 2.5 eV (Ag_2_S), 3.8 eV (Ag), and 4.6 eV (Ag_2_O); the composite (graphene/Ag–Ag_2_S) exhibits two optical gaps (∼3.28 and 4.72 eV), reflecting its multiphase nature and graphene-induced states. Methylene blue (MB) degradation follows pseudo-first-order kinetics with the strongest linearity for graphene/Ag–Ag_2_S (R^2^ ≈ 0.89–0.92). At pH 3, the hybrid achieves the highest removal efficiency (89.55% at 5 h) and the largest rate constant (k_obs = 0.5349 h^−1^). The synergy arises from assisted carrier generation in Ag, heterojunction-driven separation in Ag–Ag_2_S, and rapid electron transport/π–π adsorption on graphene, which together maximize radical formation and suppress recombination under acidic conditions.

## 1 Introduction

The global shortage of clean water continues to grow, prompting the search for science- and technology-based solutions. In this context, hybrid photocatalytic materials have become especially promising. Industrial dye effluents significantly contribute to water pollution, with the textile industry identified as the main source. Literature indicates that approximately 54% of wastewater contains dyes, emphasizing the scale of the challenge and the need for effective decolorization methods (Katheresan et al., 2018; Kaushal et al., 2021). Among common model pollutants, methylene blue (MB)—a phenothiazine dye—remains widely used for performance testing because of its persistence and known risks to environmental and human health (Khan et al., 2022). Photocatalytic dye removal occurs through light-excited charge carriers in a semiconductor. Photoexcitation generates e^−^/h^+^ pairs; their interfacial reactions produce reactive oxygen species (·OH, O_2_·^-^) that break down organic compounds into CO_2_ and H_2_O (König, 2013; Mohamadpour and Amani, 2024; Muhd Julkapli et al., 2014). Therefore, practical efficiency depends on broad or visible-light absorption, rapid charge separation and transport, minimized recombination, high surface area, and photostability. To meet these needs, recent studies have engineered plasmonic/semiconductor and carbon-hybrid architectures—such as Ag–Ag_2_S heterostructures and graphene-based composites—that improve visible-light harvesting and carrier lifetimes, resulting in higher MB degradation rates compared to their single-component versions (Chen et al., 2020; Hu et al., 2016).

Single-phase photocatalysts and adsorbents have long served as benchmarks for MB removal. Ag nanoparticles (NPs) can quickly decolorize MB under light, with recent reports showing approximately 90%–95% removal within minutes and pseudo-first-order rate constants around 10^−1^ min^−1^. This is attributed to strong LSPR-assisted charge generation and electron transfer to MB (sodium dodecyl sulfate (SDS)-capped Ag NPs achieved 92.5% in 12 min, k ≈ 0.263 min^−1^) (Almarashi et al., 2024). As a single phase, Ag_2_S (α-Ag_2_S) functions as a narrow-gap (≈1 eV) visible-light photocatalyst. Studies report efficient MB degradation under sunlight and visible light via reactive oxygen species (ROS) formation, with activity adjustable by particle size and surface states (e.g., Ag_2_S and Ni/Ag_2_S under visible light, cellulose-fiber/Ag_2_S films reaching nearly 100% MB removal in about 2 h of sunlight) (Kharlamova and Kramberger, 2022). Graphene-based materials (GO/rGO) are excellent as single-phase adsorbents for MB due to π–π interactions and electrostatic attraction; rapid uptake and high capacities are widely reported (such as quick GO adsorption, and metal-free rGO acting as an adsorbent and photocatalyst under natural light) (Siong et al., 2019). Collectively, these benchmarks clarify intrinsic roles: LSPR-enabled reduction and photocatalysis for Ag, band-gap-driven visible-light response for Ag_2_S, and high-surface-area adsorption with charge shuttling for graphene-based materials.

Ag nanomaterials with one-dimensional shapes (nanowires, nanorods) show excellent optical, electrical, thermal, and mechanical responses, mainly because of their high aspect ratios and strong LSPR effects (Yang et al., 2023). In particular, LSPR in Ag nanostructures depends heavily on size and shape: smaller particles shift the resonance to blue and increase sensitivity to the surrounding refractive index, while different shapes (spheres, triangles, rods, wires) allow precise tuning of optical responses for sensing and electrocatalysis (Ditlbacher et al., 2005; Wiley et al., 2006; Yang et al., 2023). Silver sulfide (Ag_2_S) is a chalcogenide semiconductor mainly found as monoclinic acanthite (α-Ag_2_S), which is semiconducting at room temperature, and it transforms into body-centered-cubic argentite (β-Ag_2_S) at higher temperatures, showing superionic Ag^+^ transport; nanoscale studies directly observe the α↔β transitions and confirm superionic behavior above around 450 K (Gusev and Sadovniov, 2017; Sadovnikov and Gerasimov, 2019; Simonnin et al., 2020). Electric-field–induced resistive switching in Ag/Ag_2_S junctions further demonstrates the mobility of Ag species and how conductivity is affected by phase or state changes (Billy et al., 2017; Gubicza et al., 2016; Gusev and Sadovniov, 2017). Ag_2_S has a narrow direct band gap near 1.0 eV, allowing strong absorption in the near-infrared region (Trukhanov et al., 2007; Xiang et al., 2008), making it useful for IR-active optoelectronics and photodetectors (Mubarokah et al., 2023; Wang F. et al., 2022; Zamiri et al., 2015). Its mixed ionic and electronic transport, along with high Ag^+^ conductivity, supports applications in solid-state ionics, sensors, and memory devices (Boussaida and Masrour, 2023; Shahjamali et al., 2016; Simonnin et al., 2020). When size is confined, the band gap widens; for instance, Ag_2_S nanoparticles (NPs) show, E.g., increasing from about 0.88 eV at roughly 500 nm to around 1.21 eV at approximately 60 nm, which aligns with quantum-size effects (Darvishi and Seyed-Yazdi, 2016; Sadovnikov et al., 2016). In the literature, Ag–Ag_2_S composites have also been combined with CdS to enhance visible-light activity toward dyes like MB. However, since Cd compounds are toxic and carcinogenic, these systems are not considered eco-friendly (Eswari et al., 2022; Gahramanli et al., 2024a; Gahramanli et al., 2024b; Peana et al., 2022).

Graphene, a carbon-based material considered relatively “green” and highly compatible with aqueous processing, combines extremely high electrical and thermal conductivities with excellent mechanical properties (Xin et al., 2015). Its in-plane tensile strength (∼130 GPa) and Young’s modulus (∼1 TPa) define practical limits for supporting and conductive frameworks in hybrid catalysts and membranes (Lee et al., 2008; Scarpa et al., 2009). Graphene is also chemically stable in many environments and processing methods, enabling strong device integration (Zhao et al., 2017; Zhou and Bongiorno, 2013). Due to its single-atom thickness and mass-free Dirac-fermion electronic structure, a single layer absorbs only about 2.3% of visible light but can maintain ultrahigh carrier mobility (up to ∼200,000 cm^2^ V^−1^ s^−1^ in clean suspended devices). This makes it useful for transparent electrodes and fast charge transfer in photocatalytic hybrids (Bolotin et al., 2008; Castro Neto et al., 2009; Nair et al., 2008; Zhen and Zhu, 2018).

Building on these baselines, hybrid nanostructures consistently outperform single phases by combining light harvesting, interfacial charge separation, and adsorption or dispersion. Ag–Ag_2_S heterostructures use Schottky or ohmic junctions to suppress e^−^/h^+^ recombination and extend the response into the near-infrared. Ag–Ag_2_S nanoplates showed significantly faster MB degradation than either component alone under visible light (Chen et al., 2020). Adding graphene (rGO or graphene) to Ag_2_S provides a conductive network and additional adsorption sites, resulting in better visible light MB degradation than pure Ag_2_S (Hu et al., 2016). Ternary systems further enhance these synergies: Ag/Ag_2_S/rGO composites and related Ag_2_S–oxide–graphene junctions demonstrate higher rates and better cycling stability due to efficient interfacial charge routing and dye preconcentration (e.g., Ag/Ag_2_S/rGO; Ag_2_S-sensitized NiO–ZnO) (Shafi et al., 2019). Beyond all-Ag based materials, AgNWs integrated into metal–organic frameworks (MOFs) like ZIF-8@AgNWs and various rGO-oxide hybrids also show accelerated MB removal under visible light and sunlight, underscoring the broad applicability of plasmonic–semiconductor–carbon coupling (Gahramanli et al., 2025; Negash et al., 2023).

Collectively, these advances justify exploring new Ag/Ag_2_S/graphene-type hybrids as efficient, photostable catalysts for dye removal under real-world conditions, while recognizing that testing on actual wastewater samples (beyond model MB solutions) is an essential next step for practical application (Katheresan et al., 2018; Khan et al., 2022; Mohamadpour and Amani, 2024; Muhd Julkapli et al., 2014; Pradyasti et al., 2020). The main goal of this work is to build on our previous publication by employing a novel graphene-based hybrid nanocomposite combined with Ag–Ag_2_S nanostructures for MB degradation (Gahramanli et al., 2025). The study aims to leverage graphene’s superior electron mobility, high surface area, and excellent conductivity to enable rapid charge separation and transfer during photocatalytic reactions. By integrating these benefits with the visible-light activity and semiconducting properties of Ag–Ag_2_S, the nanocomposite is expected to achieve increased photocatalytic performance. The research emphasizes developing an environmentally friendly, low-cost, and highly effective photocatalyst for environmental uses such as wastewater treatment and breaking down organic pollutants. The combined use of graphene and Ag–Ag_2_S is examined to improve stability, reusability, and light-harvesting capabilities under sustainable conditions.

## 2 Experimental part

### 2.1 Synthesis of Ag NWs

Ag NWs were synthesized using the polyol method (Alsalme et al., 2024; Gahramanli et al., 2024a). First, silicone oil was added to the reaction vessel and heated. Next, 5 mL of ethylene glycol (EG) was poured into a glass vessel and then into the silicone oil. The temperature was expected to rise to 160 °C. A mixture of 0.063 g of AgNO3 and 2 mL of, EG was stirred on a magnetic stirrer for 10 min. Then, 10 mL of CuBr2 and 15 mL of NaCl were added dropwise to the ethylene glycol over 10 min. After that, 1.5 mL of the AgNO3 mixture and 1.5 mL of PVP were slowly added dropwise to the solution over 15–20 min. The reaction was carried out for 3 h. The final solution was washed with cold water, cooled, and then 9–10 mL of acetone was added, followed by centrifugation.

### 2.2 Synthesis of Ag–Ag_2_S NWs

To synthesize Ag–Ag2S nanostructures, first, 1.33 g of Na2S is dissolved in 35 mL of distilled water to prepare a 200% solution. Then, 5 mL of the Na2S solution and 5 mL of the Ag NWs solution are taken, and both are added dropwise to the reaction vessel. The mixture is stirred for 1 h to ensure good dispersion, and the final solution is centrifuged in the same manner.

### 2.3 Synthesis of graphene nanoplates

Graphene was synthesized in a liquid medium using a microwave oven. This method stands out for its simplicity and environmental friendliness compared to other synthesis techniques. In this process, graphene layers are separated by microwave irradiation of partially exfoliated graphite in isopropyl alcohol. First, 100 mg of exfoliated graphite is dispersed in 50 mL of isopropyl alcohol. The mixture is then subjected to ultrasonic waves for 1 h. Afterward, it is filtered through filter paper, and the alcohol is absorbed with an absorbent. The mixture is then stored in a heater for 24 h to evaporate. During this time, the high evaporation capacity of isopropyl alcohol creates internal pressure between the graphite layers, weakening Van der Waals forces and causing the graphene layers to separate. This physicochemical process results in the formation of high-quality graphene layers. The obtained graphene is then dried and ground into powder.

### 2.4 Fabrication methodology of 2D/1D-graphene/Ag–Ag_2_S

To prepare the composite graphene/Ag–Ag2S nanocomposites, first, 0.008 g of Na_2_S was dissolved in 10 mL of distilled water. In parallel, 0.011 g of pre-dried Ag NWs was mixed in 10 mL of acetone, and 0.0292 g of graphene was mixed in 10 mL of isopropyl alcohol for 30 min to obtain homogeneous solutions. Then, Na_2_S solution was added dropwise to the Ag solution using a magnetic stirrer over 20–30 min. Next, 3.33 mL of each prepared solution was taken and mixed on a magnetic stirrer for 2 h. After mixing, the samples were centrifuged and washed three times with ethanol to purify them. The resulting product was dried at room temperature and powdered.

### 2.5 Photocatalysis process of MB dye by using graphene/Ag–Ag_2_S nanocatalyst

The degradation process of synthesized Ag, Ag2S NWs, and the composite material graphene/Ag–Ag2S was carried out under white light for 60, 120, 180, 240, and 300 min in different environments of MB dye using the formed graphene/Ag–Ag2S hybrid nanocatalysts in a neutral environment (pH = 6). To evaluate the degradation efficiency, graphene/Ag–Ag2S hybrid nanocatalysts were added to the prepared MB dye at a concentration of 10 ppm, and the mixed solution was exposed to white light for various durations.

To assess the degradation efficiency in an alkaline environment (pH = 10), which may have been affected by various additives (organic compounds), diluted 1M NaOH was added to the prepared 10 ppm MB dye. It was determined that the pH of the environment remained at 10. The same procedure was used to evaluate the degradation efficiency of these materials in an alkaline medium. To create an acidic medium (pH = 3), diluted 1M HCl was added to 10 ppm of MB dye. Experiments were conducted similarly, and the degradation efficiency was measured. To determine the degradation efficiency in all three environments, samples were examined at hourly intervals using UV-Vis spectroscopy. An amount of 0.01 g of each sample was used as a nanocatalyst, and the degradation of 10 mL of MB dye was studied in different pH environments. Based on the absorption spectrum observed each hour, the degradation efficiency was calculated using Equation 1 (Wang et al., 2009).

$$\frac{C_{0}-C}{C_{0}\;}*100\%=\frac{A_{0}-A}{C_{0}}$$ (1)

where C0, C, A0, and A are the concentrations and absorptions of the dye at (0) and (t) minutes under the reaction conditions, respectively. The rate constant for the photocatalytic decomposition of MB dye by hybrid nanocatalysts was analyzed using the Langmuir–Hinshelwood (LH) kinetic model and shown as Equation 2 (Sadovnikov et al., 2016).

$$\ln\frac{C_{0}}{C}=K_{obs}t$$ (2)

**FIGURE 1:**
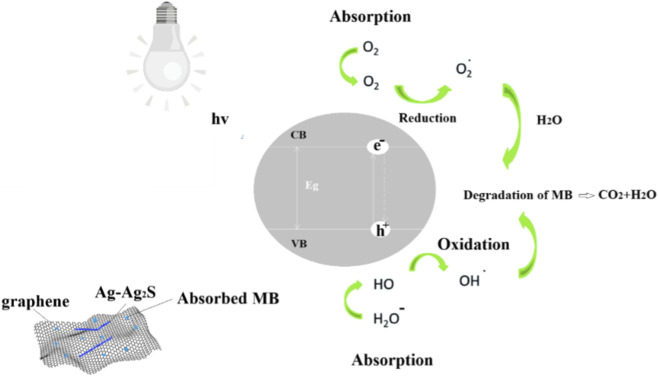
Degradation mechanism of MB by the graphene/Ag–Ag_2_S hybrid nanocatalyst.

In the Formula 1/( $K_{obs}$ t) concerning time, the derivative and intercept correspond to (C_0_)/Kc and 1/(Kc K_lh_) = $K_{obs}$ t, respectively. K_c_ is the rate constant of the surface reaction (mg L^-1^min^-1^) (Khezrianjoo and Revanasiddappa, 2012). The predicted degradation mechanism of MB by the graphene/Ag–Ag_2_S hybrid nanocatalyst is shown in Figure 1.

At Ag–Ag_2_S (and Ag_2_S–X, if a third semiconductor or carbon is present) interfaces, band offsets (or an S/Z-scheme) generate internal fields that separate photogenerated electrons and holes, so more electrons reach O_2_ to form ·O_2_^−^ and more holes produce ·OH—, both speeding up MB oxidation (Alsalme et al., 2024; Huang et al., 2024). Under visible light, Ag’s LSPR creates strong near-fields and hot electrons that inject into the nearby semiconductor (Ag_2_S or CdS, etc.), increasing reaction rates beyond either component alone; this plasmon-assisted separation and transfer are well established (Abouelela et al., 2021; Amirjani et al., 2023; Collado et al., 2018; Tatsuma et al., 2017). Because Ag_2_S has a narrow band gap (∼1 eV), combining it with plasmonic Ag and/or a wider-gap partner creates stepwise energy levels (Schottky or Z/S-scheme), allowing efficient use of visible-to-NIR light and offering multiple charge-transport pathways that pure Ag or Ag_2_S NWs lack (Liu et al., 2020; Zhang et al., 2019). Hybrid nanostructures expose more varied facets and metal/semiconductor boundaries, lowering overpotentials for surface redox steps and helping activate adsorbates, which accelerates degradation.

Graphene/rGO hybrids with Ag_2_S (and/or Ag) enhance π–π adsorption, which pre-concentrates MB on the surface, and provide ultrafast electron highways that reduce recombination and boost steady-state reactive oxygen species (ROS) flux. Graphene-modified Ag_2_S demonstrates higher visible-light activity through improved adsorption and electron transport (Hu et al., 2016; Shakoor et al., 2024). Compared to pristine Ag and Ag_2_S NWs (Balamurugan and Maruyama, 2005), these plasmonic–semiconductor hybrids (i) amplify visible-light harvesting via Ag LSPR and hot-electron transfer, (ii) create internal electric fields or Z-scheme pathways that significantly reduce e^−^–h^+^ recombination, and (iii) increase the number of interfacial active sites (and, with carbon supports, improve dye adsorption and charge mobility), collectively enhancing ·O_2_^−^/·OH flux and speeding up MB mineralization (Liu et al., 2020; Zhang et al., 2019).

### 2.6 Characterization methods

The structure of Ag, Ag2S, and graphene, both as individual components and as a composite material (graphene/Ag–Ag2S), was characterized using X-ray diffraction spectra (λ = 1.54060 Å with Ni-filtered Cu Kα radiation, Model MiniFlex 600, Rigaku Co. Ltd., Tokyo, Japan). To determine the absorbance spectrum and band gap values, as well as to investigate the degradation of MB dye over time, UV–Vis spectrophotometry (Model Specord 250 PLUS, Analytik Jena AG, Germany) was used. Chemical bonds were analyzed using FTIR spectra obtained from an IR Affinity-1 FTIR spectrometer (Shimadzu, Japan) within a wavenumber range of 400–4000 cm^−1^. The morphology of the samples was examined with a Field Emission Scanning Electron Microscope (Model JEOL JSM-7600F, JEOL Ltd., Tokyo, Japan).

### 2.7 Uncertainty propagation of the degradation percentage (%d) of MB

This section summarizes the formulas shown in Equations 3-9, compiles instrument-related uncertainty components for absorbance measurements in the 200–800 nm range (Analytik Jena SPECORD UV–Vis- PLUS series), and demonstrates the propagation of uncertainty to the percent degradation. Absorbance A at a single wavelength λ is considered a measured quantity with a standard uncertainty u(A). The degradation percentage (D%) is defined as Equation 3:

$$D\%=\frac{\left(A_{0}-A_{t}\right)}{A_{0}}\times 100=\frac{\left(1-A_{t}\right)}{A_{0}}\times 100$$ (3)

According to Guide to the Expression of Uncertainty in Measurement, the uncertainty propagation for a function F (x, y) is:

$${\;\sigma }^{2}\left(y\right)=\sum_{i}{\left(\frac{\partial F}{\partial x_{i}}\right)}^{2}{\sigma_{x_{i}}}^{2}+2\sum_{i<j}\frac{\partial F}{\partial x_{i}}\frac{\partial F}{\partial x_{j}}COV\left(x_{i}x_{j}\right)$$ (4)

Si $\sigma_{A_{0}}$ y $\sigma_{A_{t}}$ son las desviaciones típicas (incertidumbres estándar) y $COV\left(A_{0},A_{t}\right)$ su covarianza, entonces:

$$\sigma^{2}\left(D\%\right)={\left(\frac{\partial D\%}{\partial A_{0}}\right)}^{2}{\sigma_{A_{0}}}^{2}+{\left(\frac{\partial D\%}{\partial A_{t}}\right)}^{2}{\sigma_{A_{t}}}^{2}+\frac{\partial D\%}{\partial A_{0}}\frac{\partial D\%}{\partial A_{t}}COV\left(A_{0},A_{t}\right)$$ (5)

Derivadas parciales:

$$\frac{\partial D\%}{\partial A_{0}}=-\frac{100}{A_{0}},\frac{\partial D\%}{\partial A_{t}}=100\frac{A_{t}}{{A_{0}}^{2}}$$ (6)

Entonces:

$$\sigma^{2}\left(D\%\right)={\left(\frac{100}{A_{0}}\right)}^{2}\left[{\sigma_{A_{t}}}^{2}+{\left(\frac{A_{t}}{A_{0}}\right)}^{2}{\sigma_{A_{0}}}^{2}-2\frac{A_{t}}{A_{0}}COV\left(A_{0},A_{t}\right)\right]$$ (7)

The spectrometer used in the absorbance measurement is SPECORD PLUS (50/200/210/250 PLUS). Wavelength capability covers UV–Vis; the following photometric specifications apply (values from the official technical data sheet):

- Photometric accuracy (VIS, 546 nm, neutral glass filter Hellma F4): ±0.003 A
- Photometric accuracy (UV, potassium dichromate): ±0.010 A (used for UV; not used in the VIS example below)
- Photometric reproducibility (RMS at 546 nm): ≤0.0005 A
- Baseline noise at 500 nm (RMS): ≤0.0001 A
- Long-term stability at 500 nm: ±0.0005 A per hour
- Stray light: ≤0.03 %T at 220–240 nm; as low as ≤ 0.01–0.005 %T near 340 nm

Uncertainty of a single absorbance reading A. For a single absorbance reading A in the VIS, a conservative standard uncertainty can be estimated as Equation 8:

$$\sigma_{A}=\sqrt{{\sigma_{instr}}^{2}+{\sigma_{repeat}}^{2}+{\sigma_{noise}}^{2}+{\sigma_{drift}}^{2}}$$ (8)

Where:

$\sigma_{instr}$ = 0.003 A (VIS photometric accuracy),

$\sigma_{repeat}$ ≈ 0.0005 A,

$\sigma_{noise}$ ≈ 0.0001 A, and

$\sigma_{drift}$ ≈ (0.0005 A/h) × t with t the measurement span in hours.

If measurements of A_0_ and At are taken within 10 min (t = 1/6 h), then u_drift ≈0.0005 × (1/6) = 0.000083 A. Hence:

$$\sigma_{A}=\sqrt{0.003^{2}+0.0005^{2}+0.0001^{2}+0.000083^{2}}\approx 0.0030\;A$$ (9)

## 3 Results and discussions

### 3.1 Morphological analysis

**FIGURE 2:**
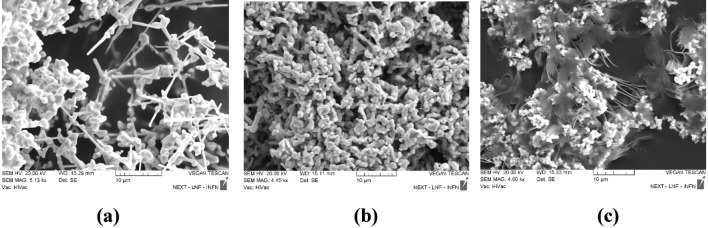
SEM images of Ag **(a)**, Ag–Ag_2_S **(b)** NWs, and graphene/Ag–Ag_2_S **(c)** composite materials.

The synthesized 1D-Ag and Ag_2_S NWs, as well as 2D/1D graphene/Ag–Ag_2_S nanocomposites, have been examined by SEM in Figure 2.

As shown in Figure 2a, pure Ag NWs were formed, with spherical small particles visible on them, as evident from the SEM images. After hybridization, Ag2S forms on the surface of the pure Ag NWs, along with Ag2O compounds in the form of crystallites (Gallardo et al., 2012) (Figure 2b). The morphology of the graphene/Ag–Ag2S nanocomposite materials is illustrated in Figure 2c. Both NWs and spherical nanoparticles are visible on the graphene surface in the Ag–Ag2S NWs nanocomposite. Therefore, the successful formation of the graphene/Ag–Ag2S hybrid nanocatalysts is confirmed by both structural and optical properties.

### 3.2 Structural analysis

**FIGURE 3:**
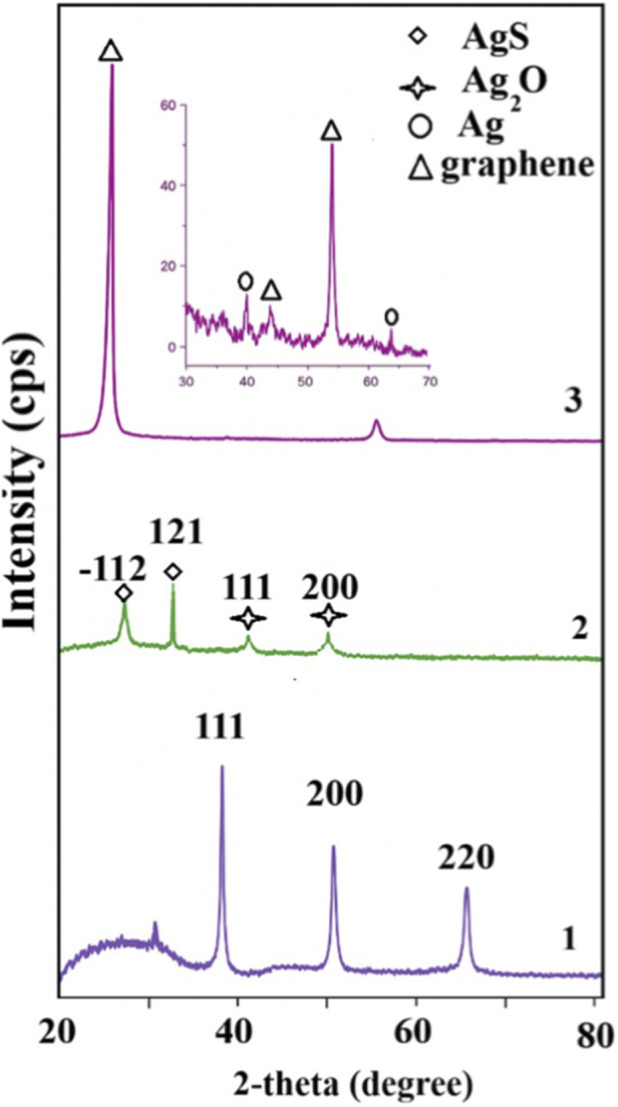
X-ray structural analysis of Ag (1), Ag–Ag_2_S (2) NWs, and graphene/Ag–Ag_2_S (3) composite materials.

X-ray structural analysis was used to determine the composition of composite materials. Figure 3 presents the individual structures of the compounds (Ag, Ag–Ag_2_S, graphene) included in the composite material.

As shown in Figure 3, three main diffraction peaks were observed at 2θ values of 38.0°, 44.8°, and 63.7°. These peaks relate to the cubic structure of Ag and match the ICDD 00-001-1167 database, as shown in the literature (Tseng et al., 2021). The peaks are indexed with the Miller indices (111), (200), and (220), respectively. The crystallite size, calculated using the Debye–Scherrer equation for each peak, is approximately 1.94 nm. The structural analysis of the Ag–Ag2S NWs after hybridization is shown in Figure 3. The diffraction pattern indicates that, after hybridization, Ag2S and Ag2O compounds form on the surface of the pure Ag NWs. These phases are labeled with Miller indices (−112) and (121), corresponding to acanthite Ag2S (JCPDS Card No. 14-0072) and cubic Ag2O (JCPDS Card No. 89-3722). The Debye–Scherrer method estimates the crystallite size at around 2.3 nm (Xiong et al., 2016). Since the structure is a 1D core-shell type, with Ag2S NWs forming on the surface of pure Ag NWs, the crystallite sizes increase compared to pure Ag due to the formation of Ag2S. Various sizes of Ag2S can develop on the surface of Ag, which can also affect the, E.g., value of the Ag–Ag2S NWs (Baghirov et al., 2024). Figure 3 presents the XRD analysis of 2D/1D structured graphene/Ag–Ag2S composite materials. The peak at 2θ = 26.4° corresponds to the (002) Miller index and has an interplanar spacing of 3.35 Å. Peaks at 2θ = 44.6° and 54.68° belong to graphene (Wang et al., 2017). Additionally, the peaks at 2θ = 38.07° and 64.36° are diffraction peaks from Ag NWs. No characteristic peaks of Ag2S are observed in the recorded diffraction pattern, likely because the characteristic peaks of Ag2S are weaker compared to those of other components.

### 3.3 Optical properties

**FIGURE 4:**
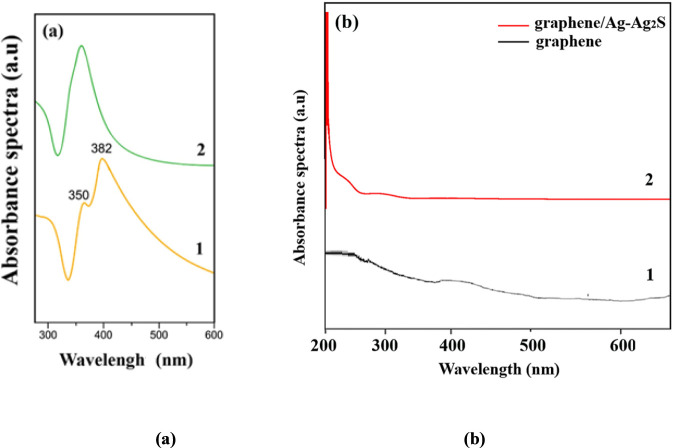
Absorbance spectrum of **(a)** Ag (1), Ag–Ag_2_S (2) NWs, and **(b)** graphene (1), and graphene/Ag–Ag_2_S (2).

To study the absorption spectra of the samples (Figure 4), each was studied by UV-Vis spectroscopy. The absorption spectrum of Ag (1), Ag–Ag_2_S (2) NWs in Figure 4a, and graphene nanoplates (1), and graphene/Ag–Ag_2_S (2) composite materials in Figure 4b was studied.

The absorption spectrum of the synthesized Ag NWs displays two characteristic peaks at 350 nm and 382 nm (Cirit et al., 2024; Gebeyehu et al., 2017). The peak at 350 nm is due to the out-of-plane quadrupole resonance of the Ag NWs, while the more intense peak at 380 nm results from the out-of-plane dipole resonance (Gao et al., 2005). As shown in the absorbance spectra of Ag–Ag_2_S NWs (Figure 4a), the two shoulder-like peaks typical of pure Ag NWs are missing (Zhang et al., 2017). With increasing Ag_2_S coverage during hybridization, the wires become thicker and their surfaces more fully coated with Ag_2_S. During sulfidation, sulfide ions (S^2-^) diffuse inward most efficiently at the surface—where their concentration is highest—and less effectively with depth due to limited penetration. This process reduces the electron concentration near the surface and suppresses SPR.

Consistent with previous reports on Ag NWs (∼350/∼380 nm LSPR) and their sulfidized versions, Ag–Ag_2_S NWs no longer show the ∼350 nm shoulder. The Ag_2_S shell increases dielectric loss and boosts interfacial non-radiative damping, causing a broadened, merged band instead of two separate Ag modes (Gebeyehu et al., 2017; Jiang et al., 2017; Wang S. et al., 2022). Mechanistically, the Ag_2_S shell (i) raises the local refractive index and introduces lossy dielectric damping (higher extinction coefficient), which broadens and weakens the Ag LSPR; (ii) adds surface/Ohmic and interfacial charge transfer pathways that dissipate plasmon energy non-radiatively; and (iii) changes morphology/roughness, disrupting coherent transverse modes. Collectively, these effects suppress the distinct shoulder observed in bare Ag NWs. Reports on Ag@Ag_2_S systems show the same replacement of sharp Ag peaks with broadband absorption, and general LSPR theory also predicts peak broadening and attenuation when a metal is coated with a higher-index, absorptive shell (Jiang et al., 2017; Li et al., 2015; Mcoyi et al., 2025; Wang et al., 2012). The frequency of plasmon oscillations depends on the charge carrier concentration, as shown by Equation 10, which relates plasmon oscillations to charge carrier density (Zhu et al., 2015).

$$\omega_{p}=\sqrt{\frac{N_{h}e^{2}}{\varepsilon_{0}m_{h}}}$$ (10)

Where e is the electron charge, *ε*0 is the permittivity of free space, mh is the hole effective mass (approximately 0.8 m0, where m0 is the electron mass), and Nh is the free hole density. Equation 10 shows that plasmon oscillations are influenced by charge carrier concentration. In other words, the frequency of plasmon oscillations varies with concentration. Because there are many free-charge carriers on the metal’s surface, the frequency of plasmon oscillations is also high. When the free charge carriers on the metal’s surface are replaced by a different concentration in the semiconductor, the frequency of plasmon oscillations decreases. The decrease in electron concentration reduces the SPR frequency. As Ag_2_S is less conductive than metallic Ag, the formation of Ag_2_S causes a decrease in the SPR frequency, shifting the plasmon band toward longer wavelengths—a red shift in the UV–Vis absorption spectrum. After hybridization, a red shift is observed in the absorption peaks (Gahramanli et al., 2024c).

Thus, based on the results obtained from UV-Vis spectroscopy, it can be said that both Ag and Ag–Ag_2_S NWs were successfully synthesized. Since oxygen is dissolved in water during hybridization and the reaction is carried out in an atmospheric environment, oxygen is not removed from the reaction environment (it is not carried out in a nitrogen environment), Ag_2_O is formed as a result of the oxidation of Ag, which is evident from both XRD and UV-Vis. results.

Figure 4b shows the UV-Vis spectra of graphene. The absorption spectrum of graphene displays a typical absorption curve reported in the literature (Gahramanli et al., 2024c) for graphene nanosheets. According to the literature, weak absorption in the range of 320–680 nm is characteristic of graphene nanosheets. Figure 4b shows the absorption spectra of graphene/Ag–Ag2S nanocomposites. As seen in the spectrum, the main absorption region of the nanocomposites is between 200 and 250 nm. This corresponds to the π–π* electronic transitions of graphene, related to its π-electron system (Uzek, 2024). Such transitions are characteristic of the ultraviolet range, common in aromatic structures, especially in carbon-based materials, and are also observed in graphene oxide or functionalized graphene structures (Dreyer et al., 2010).

Conversely, the Ag and Ag_2_S nanostructures in the nanocomposite also influence the spectrum. Ag NWs can absorb in the visible and near-UV regions due to SPR. Typically, the SPR peak for Ag nanostructures is between 350 and 450 nm. However, because of the interaction of Ag–Ag_2_S NWs with graphene and the formation of solid solutions or interactions of Ag with Ag_2_S, this absorption band shifts to a lower wavelength — 250 nm (blue shift) (Liz-Marzán, 2006). After 250 nm, absorption drops sharply and stays nearly constant for wavelengths above 300 nm. This occurs because the nanocomposite is mainly active in the UV region and absorbs minimally in the visible spectrum.

**FIGURE 5:**
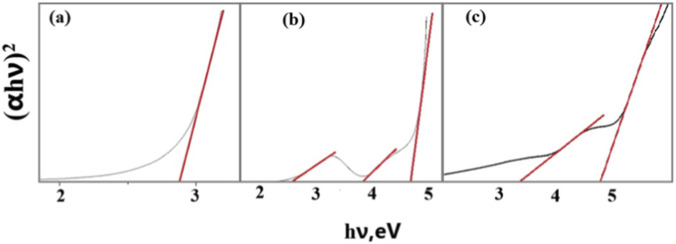
Determination of the bandgap value of Ag **(a)**, Ag–Ag_2_S NWs **(b)**, and graphene/Ag–Ag_2_S hybrid nanocomposite materials **(c)**.

In Figure 5, Tauc curves are presented to determine the band gap value for Ag (a), Ag_2_S (b) NWs, as well as graphene/Ag–Ag_2_S (c) composite materials.

By extrapolating the obtained straight line, the band gap of Ag NWs (Figure 5a) was found to be 2.9 eV. Although pure bulk Ag nanostructures do not have a band gap, Ag nanostructures exhibit a certain band gap due to quantum effects. This is explained by the increase in SPR and size effects, especially for 1D structures (NWs) (Jana et al., 2001). Due to hybridization, the band gap value tripled (Figure 5b). Consequently, after hybridization, Ag_2_O was also formed in the composite material. Based on the Tauc plot analysis, the, E.g., values after hybridization were approximately 2.5 eV for Ag_2_S, 3.8 eV for Ag NWs, and 4.6 eV for Ag_2_O.

Two different straight-line extrapolations of the composite material were obtained from the (αhν)^2^ ∼ hν dependence of graphene/Ag–Ag2S in Figure 5c. Using these lines, two different band gap energies of 3.28 eV and 4.72 eV were determined. This reflects the multiphase and multicomponent nature of the material. The graphene component can create local states that influence optical conductivity and electronic transitions (Eda and Chhowalla, 2010). Therefore, 3.28 eV is linked to low-energy transitions resulting from the interaction between nanoscale Ag_2_S and graphene. The other, E.g., value (4.72 eV) is due to changes in plasmon resonance and carrier mobility caused by incorporating graphene into the Ag-based structure, leading to broadening of energy levels. This suggests that the 2D/1D graphene/Ag–Ag2S nanocomposites have potential applications in photocatalysis.

### 3.4 FTIR analysis

**FIGURE 6:**
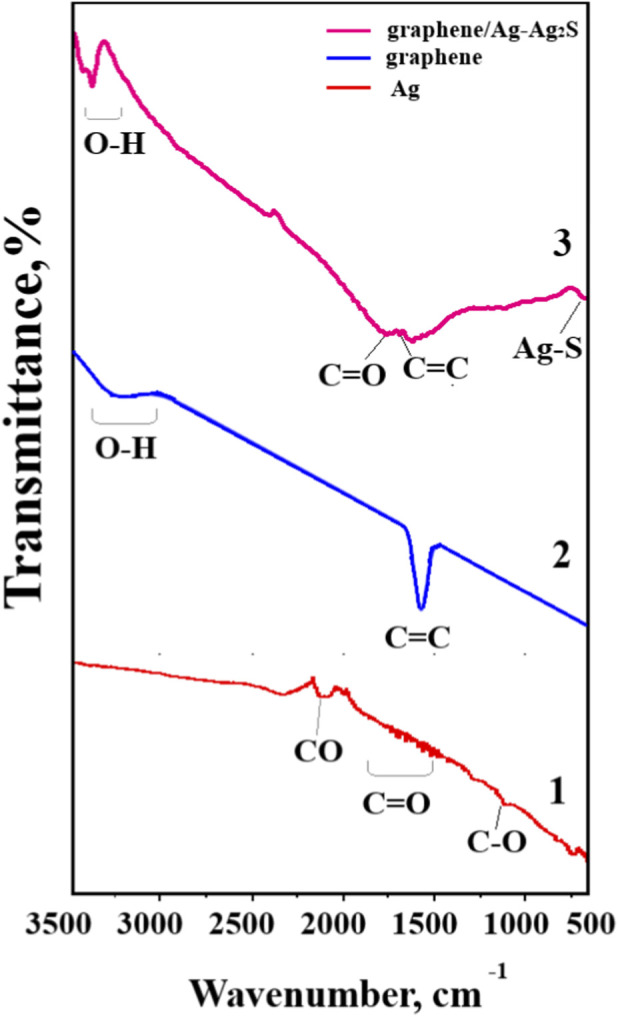
FTIR spectrum of graphene/Ag–Ag_2_S nanocomposite materials: 1-Ag NWs; 2-graphene; 3-graphene/Ag–Ag_2_S.

FTIR analysis was performed to determine the chemical bonds between elements in graphene/Ag–Ag_2_S composite materials, and the results are shown in Figure 6.

Figure 6 shows the FTIR spectrum of the synthesized materials. In Figure 6, the spectrum of pure Ag NWs displays 2200–2000 cm^−1^ – CO vibrations adsorbed on the Ag surface (Wang et al., 2025), 1660 cm^−1^ – weak vibrations in PVP are slightly shifted due to the carbonyl group C=O, which coordinates with the silver surface (Kumar et al., 2017). The 1100–1050 cm^−1^ peak corresponds to C–O stretching, related to PVP and residual, EG. These results indicate that the surface of the Ag NWs is coated with PVP. In Figure 6, the spectrum shows 3400–3200 cm^−1^ – O–H stretching of adsorbed water, and 1630–1580 cm^−1^ – weak aromatic C=C skeleton vibrations characteristic of pure graphene (Dreyer et al., 2010). The IR spectrum of graphene/Ag–Ag2S nanocomposites is shown in Figure 6. A broad absorption band around 3552 cm^−1^, 3476 cm^−1^, and 3415 cm^−1^ can be attributed to O–H stretching vibrations (generally within 3300–3600 cm^−1^), due to adsorbed moisture or hydroxyl groups in the precursor graphite structure (Hadi et al., 2018) (Wang et al., 2025). A notable absorption peak at 1621 cm^−1^ corresponds to the C=C stretching vibration of the sp^2^-hybridized carbon atoms in the hexagonal graphene lattice (Ickecan et al., 2017) (Kumar et al., 2017).

A weaker band at 1696 cm^−1^ is assigned to the C=O stretching vibration, characteristic of PVP chains adsorbed onto the surface of Ag NWs (Shokri Doodeji et al., 2024) (Dreyer et al., 2010). The Ag–S vibrational mode appears as a peak at 537 cm^−1^, which is also often associated with Ag–O bonds, indicating the presence of both Ag_2_S and Ag_2_O phases (Pradheesh et al., 2020; Zamiri et al., 2015) (Hadi et al., 2018; Zamiri et al., 2015; Zamiri et al., 2015). Overall, the main FTIR signals are primarily attributed to graphene, reflecting its high surface area and abundance of functional groups. In contrast, signals related to silver compounds (Ag_2_S and Ag_2_O) are weaker. These FTIR results align with the XRD and SEM analyses, confirming that oxidation took place during the sulfidation process, as shown by the presence of oxygen-containing vibrational modes.

### 3.5 Photodegradation of MB by graphene/Ag–Ag_2_S nanocatalyst: UV–Vis absorbance evolution, degradation efficiency, and kinetic rate analysis

**FIGURE 7:**
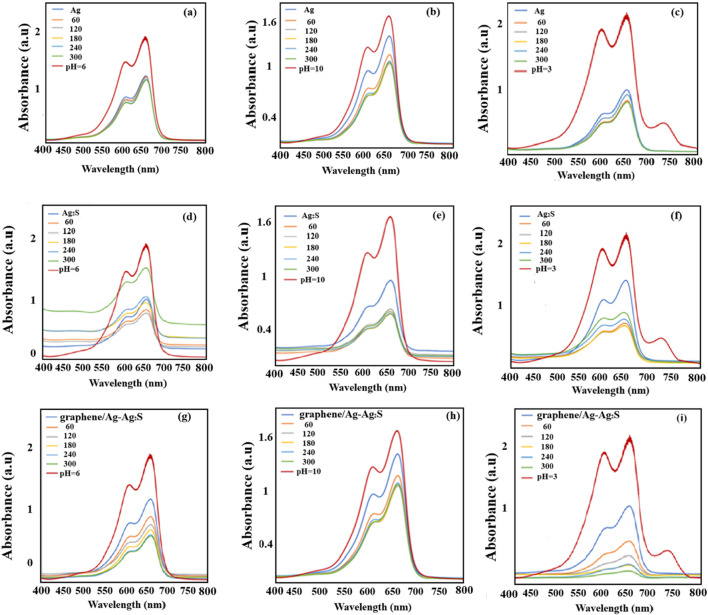
Absorption spectra of MB dye with Ag NWs, Ag–Ag_2_S NWs, and graphene/Ag–Ag_2_S **(a,d,g)** neutral medium (pH = 6); **(b,e,h)** alkaline medium (pH = 10); **(c,f,i)** acidic medium (pH = 3).

Both the separately prepared Ag NWs and the Ag–Ag2S NWs obtained after hybridization, as well as the composite graphene/Ag–Ag2S hybrid nanocatalysts, were used to perform photocatalysis of MB dye in various environments with pH levels of 3 (acidic), 6 (neutral), and 10 (alkaline) for 60–300 min. To assess degradation efficiencies, absorption spectra were recorded every 60 min using UV-Vis spectroscopy. Figure 7 displays the absorption spectra collected during the photocatalysis process with Ag NWs for neutral (Figure 7a), alkaline (Figure 7b), and acidic (Figure 7c), with Ag–Ag2S NWs for neutral (Figure 7d), alkaline (Figure 7e), and acidic (Figure 7f), and with graphene/Ag–Ag2S nanocomposite materials for neutral (Figure 7g), alkaline (Figure 7h), and acidic (Figure 7i) media.

**FIGURE 8:**
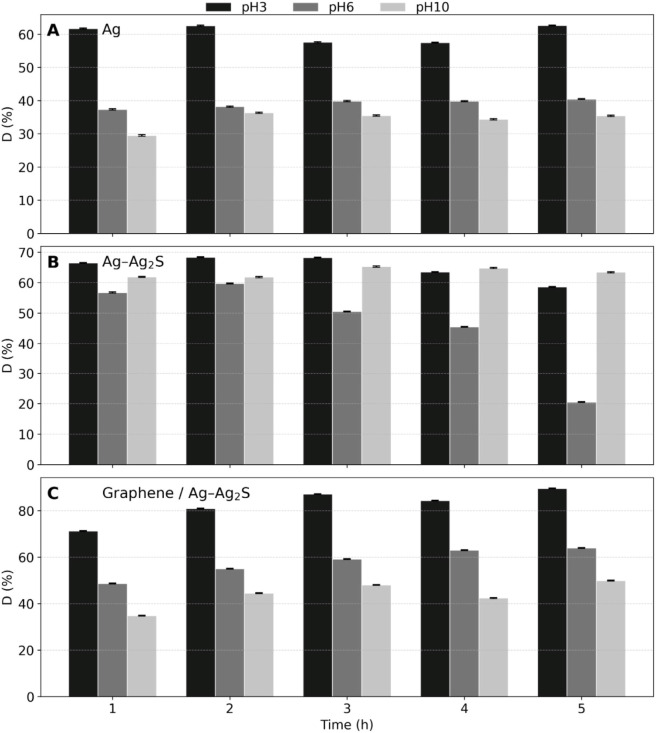
The average degradation efficiency of MB dye by Ag **(A)**, Ag–Ag_2_S **(B)** NWs, and **(C)** graphene/Ag–Ag_2_S nanocatalysts.

According to Equation 1, the average degradation efficiencies for each nanocatalyst at each pH –(Ag NWs -Figure 8A, Ag–Ag_2_S NWs-Figure 8B, and graphene/Ag–Ag_2_S -Figure 8C) are presented in Figure 8 and summarized in Table 1.

**TABLE 1:** The average degradation percentages (%) of MB dye by Ag, and Ag–Ag_2_S, graphene/Ag–Ag_2_S NWs. All the uncertainties were found to be less than 1%.

​	Ag					
Time (h)	pH = 3		pH = 6		pH = 10	
	*D%*	*σD%*	*D%*	*σD%*	*D%*	*σD%*
1	61.7301	±0.1516	37.3364	±0.1853	29.5124	±0.2204
2	62.5844	±0.1512	38.1740	±0.1846	36.3396	±0.2135
3	57.5818	±0.1538	39.8597	±0.1833	35.4990	±0.2144
4	57.4827	±0.1538	39.8231	±0.1833	34.3821	±0.2155
5	62.6363	±0.1511	40.4670	±0.1828	35.4329	±0.2144

​	Ag–Ag_2_S					
Time (h)	pH = 3		pH = 6		pH = 10	
	*D%*	*σD%*	*D%*	*σD%*	*D%*	*σD%*
1	66.5014866	±0.1493156	56.7846	±0.1711	61.9310676	±0.19274854
2	68.3845391	±0.14849027	59.7791	±0.1693	61.871022	±0.19278705
3	68.2759923	±0.14853667	50.5706	±0.1752	65.3116368	±0.19066691
4	63.5612818	±0.15068957	45.4612	±0.1789	64.85529	±0.1909379
5	58.6530747	±0.15320793	20.5581	±0.2006	63.4442176	±0.19179569

​	Graphene/ Ag–Ag_2_S					
Time (h)	pH = 3		pH = 6		pH = 10	
	*D%*	*σD%*	*D%*	*σD%*	*D%*	*σD%*
1	71.2115	±0.1473	48.7017066	±0.17651033	34.8204636	±0.2150232
2	80.9241	±0.1441	54.9785363	±0.17223438	44.5238381	±0.2059998
3	87.1348	±0.1427	59.1456392	±0.16965267	48.0184941	±0.20302058
4	84.3268	±0.1433	63.0300492	±0.16744072	42.4042272	±0.20787887
5	89.5512	±0.1424	63.9357135	±0.16695283	49.8979224	±0.20148148

MB degradation was highest in acidic media. For Ag, it was 62.63% at pH 3 (5 h) versus 40.46% at pH 6 and less than or equal to 36.33% at pH 10 after 2 h. For Ag–Ag_2_S NWs, the maximum was 68.27% at pH 3 (3 h; with no further change afterward), 59.77% at pH 6 (2 h), and 65.31% at pH 10 (3 h). For graphene/Ag–Ag_2_S, it was 89.55% at pH 3 (5 h), 63.94% at pH 6, and 49.90% at pH 10 (5 h). In acidic conditions, (i) valence-band holes are stronger oxidants that efficiently generate ·OH/·O_2_^−^, accelerating N-demethylation and ring-opening of cationic MB; (ii) Ag_2_S captures visible/NIR light and, with Ag LSPR, enhances near-surface field intensity, boosting ROS formation; and (iii) graphene sheets in the hybrid facilitate e^−^ transport, reduce e^−^/h^+^ recombination (PL quenching/ higher photocurrent), and sustain ROS flux—together explaining the highest rate at pH 3 (Alaizeri et al., 2023; Mubarokah et al., 2023; Shokri Doodeji et al., 2024).

At neutral pH, activity decreases (Ag 40.46%; graphene/Ag–Ag_2_S 63.94%) as hole oxidizing ability diminishes and adsorption becomes more influenced by surface charge. MB is mainly cationic, and its adsorption depends on the catalyst’s pH at the point of zero charge. Negative surfaces favor MB^+^ uptake, while protonated surfaces hinder electrostatic adsorption, leading to a lower local dye concentration at active sites and slower kinetics (Jaramillo-Fierro and Cuenca, 2024; Fito et al., 2023) (Alaizeri et al., 2023; Pradheesh et al., 2020). In alkaline conditions, two opposing effects occur. First, higher [OH^−^] levels can promote ·OH formation on some photocatalysts; second, Ag-based surfaces undergo pH-dependent transformations (transient Ag_2_O/Ag–O species formation/dissolution; surface restructuring) and stronger product adsorption/passivation, which can stall progress (Ag stabilizing after 120 min).

The Ag–Ag_2_S heterojunction partially compensates for these losses by enhancing charge separation (visible/NIR harvesting and interfacial band alignment), resulting in a relatively high efficiency of 65.31% at 180 min. Graphene further stabilizes reaction rates by improving adsorption (π–π interactions and van der Waals forces) and charge transport. However, excessive OH^−^ can still decrease net ROS availability on the surface and compete for active sites, which aligns with the lower efficiency of 49.90% at pH 10 (Mubarokah et al., 2023; Shokri Doodeji et al., 2024; Jaramillo-Fierro and Cuenca, 2024). Overall, acidic conditions maximize hole-driven oxidation and plasmon-assisted ROS generation, while neutral and alkaline environments introduce limitations related to adsorption and surface chemistry. The graphene/Ag–Ag_2_S hybrid best alleviates these issues through improved MB uptake and reduced recombination (Alaizeri et al., 2023; Jaramillo-Fierro and Cuenca, 2024) (Pradheesh et al., 2020; Shokri Doodeji et al., 2024).

**FIGURE 9:**
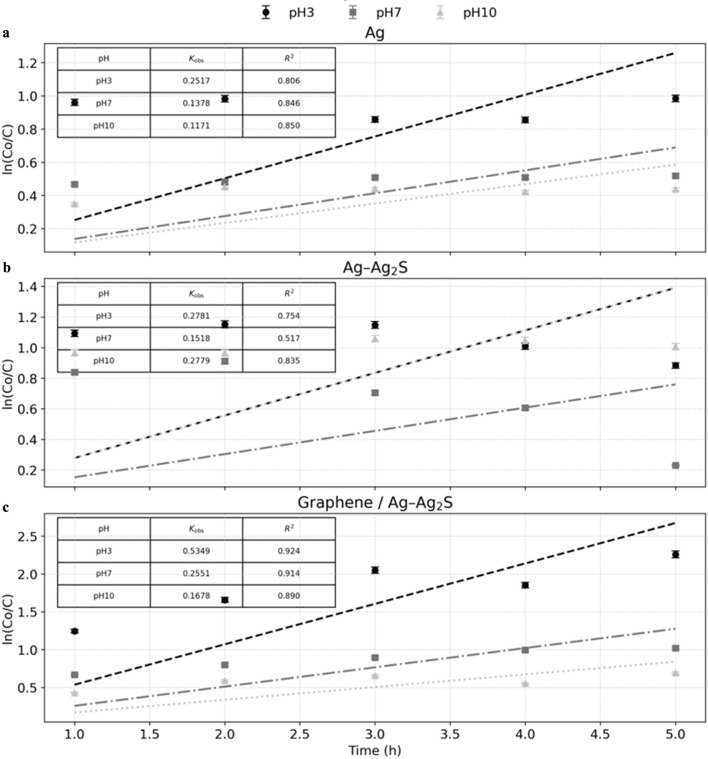
Photocatalytic kinetic rate constants of MB by nanocatalysts **(a)** Ag NWs; **(b)** Ag–Ag_2_S; **(c)** graphene/Ag–Ag_2_S nanocatalysts.

Photocatalytic kinetic rate constants (k_oβs_, min^−1^) and the coefficient of determination (R^2^) for each nanocatalyst across each medium (Ag NWs -Figure 9a, Ag–Ag_2_S NWs-Figure 9b, and graphene/Ag–Ag_2_S -Figure 9c) are presented in Figure 9.

Across all catalysts, ln(C_0_/C) increases roughly linearly with time, supporting a pseudo-first-order model. The best linearity is observed for the graphene/Ag–Ag_2_S hybrid (R^2^ ≈ 0.89–0.92), while moderate fits are seen for pure Ag (R^2^ ≈ 0.81–0.85). Ag–Ag_2_S at pH 7 shows noticeable deviation (R^2^ = 0.517). Rate constants (kobs, h^-1^) reveal a clear activity ranking that depends on pH: under acidic conditions (pH 3), graphene/Ag–Ag_2_S dominates with 0.5349, exceeding both Ag–Ag_2_S (0.2781) and Ag (0.2517), resulting in approximately 2.1 times higher rates than Ag and Ag–Ag_2_S. At neutral pH 7, the same order applies—0.2551 (graphene/Ag–Ag_2_S) > 0.1518 (Ag–Ag_2_S) > 0.1378 (Ag)—with about 1.9 times higher rates compared to Ag. In alkaline media (pH 10), however, Ag–Ag_2_S (0.2779) outperforms both graphene/Ag–Ag_2_S (0.1678) and Ag (0.1171), achieving roughly 2.4 times and 1.6 times higher rates, respectively. Overall, an acidic environment generally speeds up degradation for all materials. The graphene-modified hybrid delivers the highest kinetics at pH 3, and Ag–Ag_2_S exhibits a notable performance boost at pH 10.

### 3.6 Limitations and perspectives

This study establishes the synthesis–structure–activity relationship of a graphene/Ag–Ag_2_S hybrid toward methylene blue under controlled conditions, but it does not experimentally resolve the full degradation mechanism. The mechanistic rationale advanced here—plasmonic excitation in Ag, photoexcitation in Ag_2_S, and graphene-mediated charge extraction with π–π enrichment of MB—remains a proposed pathway supported by literature rather than by new, dedicated diagnostics. In future work we will validate the dominant reactive oxygen species and charge-transfer sequence using dye-loaded spectroscopy (pre/post FT-IR or Raman), radical scavenger assays and ESR spin-trapping, and carrier-dynamics probes such as steady-state or time-resolved photoluminescence and electrochemical impedance. By separating adsorption from photoreaction and correlating spectral changes with kinetic signatures, these measurements will convert the present mechanistic proposal into direct experimental evidence.

The present report does not include reusability testing or long-term stability analysis. The study was performed on a small test batch synthesized to confirm composition, phases, and baseline photocatalytic response; this limited mass was insufficient to support statistically powered multi-cycle experiments alongside characterization. We therefore refrain from making durability claims. Subsequent work will scale the synthesis to enable repeated-use studies under identical conditions with triplicate measurements, report mean ± standard deviation for degradation efficiencies and apparent rate constants, annotate kinetic fits with *R*2, and examine post-reaction integrity by XRD, XPS, and FT-IR. Silver leaching will be quantified to verify heterogeneous operation, and any efficiency decay will be mapped to specific failure modes such as photocorrosion, surface fouling, or phase evolution.

Finally, we did not evaluate performance in industrial wastewater matrices. This is intentional to isolate intrinsic activity and avoid matrix-dependent artifacts in an initial materials report. Real-sample validation will be followed by testing representative effluents alongside standard MB solutions to quantify matrix effects from pH, ionic strength, turbidity, and co-contaminants. Spike-and-recovery protocols will be used to assess accuracy, total organic carbon or chemical oxygen demand will be monitored to gauge mineralization beyond decolorization, and catalyst integrity and leaching will be checked after treatment. These steps will translate the present proof-of-concept into a deployable treatment strategy while preserving the rigor of controlled benchmarking.

## 4 Conclusion

A robust 2D/1D graphene/Ag–Ag_2_S photocatalyst was successfully synthesized and validated through complementary characterization methods. SEM revealed a core–shell Ag–Ag_2_S structure on 1D NWs decorating 2D graphene. XRD confirmed the presence of fcc-Ag along with acanthite Ag_2_S and Ag_2_O. UV–Vis results showed LSPR suppression and broadening upon shelling, with π–π* dominated absorption in the hybrid, as seen in the UV-Vis spectrum and Tauc plots indicating multiphase band structures (E.g., ∼3.28/4.72 eV), which are advantageous for interfacial charge separation. FTIR verified Ag–S/Ag–O bonding alongside graphene signatures. These structure–property relationships explain the photocatalytic performance: (i) Ag LSPR supplies hot carriers; (ii) Ag–Ag_2_S heterojunctions promote charge separation; and (iii) graphene enhances electron transport and dye adsorption. As a result, the hybrid achieved the fastest MB degradation in acidic media (pH 3: kobs = 0.5349 h^−1^; 89.55% in 5 h), surpassing both Ag–Ag_2_S and Ag. Performance was moderated at neutral pH with the same activity order, while alkaline conditions favored Ag–Ag_2_S (kobs = 0.2779 h^−1^) as surface chemistry and site competition reduced graphene’s advantage. The characterization confirms successful hybrid formation and explains its pH-responsive, high-efficiency dye removal, positioning graphene/Ag–Ag_2_S as a tunable platform for wastewater remediation, with Ag–Ag_2_S alone recommended for alkaline streams.

## Data Availability

The original contributions presented in the study are included in the article/supplementary material, further inquiries can be directed to the corresponding author.
